# DELIRIUM TREMENS IN A MIDDLE-AGED MALE ICU PATIENT

**DOI:** 10.51894/001c.123099

**Published:** 2024-08-30

**Authors:** Yousef Alsmairat

**Affiliations:** 1 Internal Medicine Detroit Medical Center Sinai-Grace Hospital

## 84

### INTRODUCTION

More than 50% of those with a history of alcohol use disorder can exhibit alcohol withdrawal symptoms when discontinuing their alcohol use. Only a few patients exhibit symptoms of severe alcohol withdrawal characterized by profound confusion and cardiovascular collapse. This is defined as delirium tremens (DTs). DTs symptoms continue for 3-4 days and typically resolve after five days.

### CASE DESCRIPTION

A 43-year-old male presented to the emergency department with dyspnea, productive cough, fever, chills, and generalized weakness for four days. Vital signs during admission showed elevated blood pressure, tachycardia, and increased oxygen requirements. Lab values revealed elevated lactic acid, mild hyponatremia, hypokalemia, metabolic acidosis, and mildly elevated liver enzymes. The alcohol level was at 10. A CT scan of the chest revealed pneumonia in the right lower and middle lobes. Antibiotic treatment was started for aspiration pneumonia. The patient had his last alcoholic drink 4 days admission. Due to profound agitation and confusion, the patient required intubation and ICU admission and was treated for DTs. All other possible causes of confusion were excluded through labs and physical exams. The patient was then diagnosed and treated as DTs and CIWA protocol was initiated. On day 5 of admission, the patient continued to show signs of delirium tremens while intubated and required high levels of sedation. The patient was on midazolam and fentanyl drip and was having episodes of recurrent agitation, tremors, tachycardia, bouts of sweating, and increased anxiety, especially during spontaneous breathing trials. This led to multiple failed extubation trials despite improvement in pneumonia and receiving sufficient prophylactic doses of chlordiazepoxide with a total of 75 mg a day. The patient continued to have these episodes 11 days after his last alcoholic drink and 8 days after admission.

### DISCUSSION

This case demonstrates a rare case of prolonged DTs that continued beyond the usual 5-day period despite adequate benzodiazepines and Versed Drip that led to multiple failed spontaneous breathing trials and delayed extubation. It was diagnosed after excluding all other possible diagnoses mimicking DTs. More extensive studies are required to highlight and define the causes, management, and complications of prolonged DTs.

